# Supplementary material for: Biology exams rarely use visual models to engage higher-order cognitive skills
Source: PLoS One. 2025 Jul 2;20(7):e0317077. doi: 10.1371/journal.pone.0317077 (PMC12221023; doi:10.1371/journal.pone.0317077)
Supplement: S1 Table — (DOCX) [file pone.0317077.s002.docx]

**Biology exams rarely use visual models to engage higher-order cognitive skills**

Crystal Uminski, Christian Cammarota, Brian A. Couch, L. Kate Wright, Dina L. Newman

**S1 Table: Institutional Carnegie classifications**

| **Institution classification** | **Number of institutions** |
| --- | --- |
| Associate’s | 16 |
| Baccalaureate | 15 |
| Master’s | 13 |
| Doctoral | 22 |
| **Total** | 66 |
| Institutional classifications are based on Carnegie classifications (Indiana University Center for Postsecondary Research 2021). | |
