# Supplementary material for: Biology exams rarely use visual models to engage higher-order cognitive skills
Source: PLoS One. 2025 Jul 2;20(7):e0317077. doi: 10.1371/journal.pone.0317077 (PMC12221023; doi:10.1371/journal.pone.0317077)
Supplement: S2 Table — (DOCX) [file pone.0317077.s003.docx]

**Biology exams rarely use visual models to engage higher-order cognitive skills**

Crystal Uminski, Christian Cammarota, Brian A. Couch, L. Kate Wright, Dina L. Newman

**S2 Table: Self-reported teaching experience of undergraduate biology instructors**

| **Teaching experience as instructor of record** | **Number of instructors** |
| --- | --- |
| 0-1 year | 3 |
| 2-5 years | 14 |
| 6-10 years | 19 |
| 11-15 years | 13 |
| 16-20 years | 7 |
| 21-25 years | 8 |
| > 25 years | 2 |
