# Supplementary material for: Biology exams rarely use visual models to engage higher-order cognitive skills
Source: PLoS One. 2025 Jul 2;20(7):e0317077. doi: 10.1371/journal.pone.0317077 (PMC12221023; doi:10.1371/journal.pone.0317077)
Supplement: S3 Table — (DOCX) [file pone.0317077.s004.docx]

**Biology exams rarely use visual models to engage higher-order cognitive skills**

Crystal Uminski, Christian Cammarota, Brian A. Couch, L. Kate Wright, Dina L. Newman

**S3 Table: Linear mixed model predicting the point value of items with visual models**

| **Fixed effects** | **β** | **Standard Error** | ***df*** | ***t*** | ***p*** |
| --- | --- | --- | --- | --- | --- |
| Intercept | 4.97 | 1.52 | 63.11 | 3.26 | 0.0018 |
| Contains model | 0.41 | 0.092 | 2618.49 | 4.43 | < .0001 |
| Model: Item point value ~ Contains model + (1\|instructor) Reference group: No model R^2^ = 0.98  Item point values were normalized to a scale of 0 – 100 for each instructor. | | | | | |
